# Carbohydrate Metabolism Is Essential for the Colonization of *Streptococcus thermophilus* in the Digestive Tract of Gnotobiotic Rats

**DOI:** 10.1371/journal.pone.0028789

**Published:** 2011-12-22

**Authors:** Muriel Thomas, Laura Wrzosek, Leila Ben-Yahia, Marie-Louise Noordine, Christophe Gitton, Didier Chevret, Philippe Langella, Camille Mayeur, Claire Cherbuy, Françoise Rul

**Affiliations:** 1 Commensal and Probiotics-Host Interactions Laboratory, INRA, UMR1319 Micalis, Jouy-en-Josas, France; 2 Peptides and Bacterial Communication Laboratory, INRA, UMR1319 Micalis, Jouy-en-Josas, France; 3 PAPPSO (Plateforme d'Analyse Protéomique de Paris Sud-Ouest) proteomic platform, INRA, UMR1319 Micalis, Jouy-en-Josas, France; Paris Institute of Technology for Life, Food and Environmental Sciences, France

## Abstract

*Streptococcus thermophilus* is the archetype of lactose-adapted bacterium and so far, its sugar metabolism has been mainly investigated *in vitro*. The objective of this work was to study the impact of lactose and lactose permease on *S. thermophilus* physiology in the gastrointestinal tract (GIT) of gnotobiotic rats. We used rats mono-associated with LMD-9 strain and receiving 4.5% lactose. This model allowed the analysis of colonization curves of LMD-9, its metabolic profile, its production of lactate and its interaction with the colon epithelium. Lactose induced a rapid and high level of *S. thermophilus* in the GIT, where its activity led to 49 mM of intra-luminal L-lactate that was related to the induction of mono-carboxylic transporter mRNAs (SLC16A1 and SLC5A8) and p27^Kip1^ cell cycle arrest protein in epithelial cells. In the presence of a continuous lactose supply, *S. thermophilus* recruited proteins involved in glycolysis and induced the metabolism of alternative sugars as sucrose, galactose, and glycogen. Moreover, inactivation of the lactose transporter, LacS, delayed *S. thermophilus* colonization. Our results show i/that lactose constitutes a limiting factor for colonization of *S. thermophilus,* ii/that activation of enzymes involved in carbohydrate metabolism constitutes the metabolic signature of *S. thermophilus* in the GIT, iii/that the production of lactate settles the dialogue with colon epithelium. We propose a metabolic model of management of carbohydrate resources by *S. thermophilus* in the GIT. Our results are in accord with the rationale that nutritional allegation *via* consumption of yogurt alleviates the symptoms of lactose intolerance.

## Introduction

*Streptoccocus thermophilus (S. thermophilus)* is a dairy bacterium consumed by Humans for centuries. *S. thermophilus* is in the European Qualified Presumption of Safety list of food bacteria and is a generally recognized as a safe species (GRAS status); it has a long documented history of safe use in food and its genome is devoided of potential virulence functional genes [1], [2]. *S. thermophilus* is found in numerous cheeses and is one of the two bacteria in yogurt with its obligate partner *Lactobacillus delbrueckii* sp. *bulgaricus (L. bulgaricus)*. Its high performance in fermenting milk and its resistance to elevated temperatures are two essential properties that accounted for a large utilization of strains of the species *S. thermophilus* in dairy industry. In addition, *S. thermophilus* is also recognized as a probiotic and, therefore, used to promote health [3], [4], [5], [6].

Recently, a general claim on yogurt on aiding lactose digestion has been accepted by the European Food Safety Authority. The latter considers that a cause and effect relationship is established between the consumption of live yogurt culture and improvement of lactose digestion in individuals with lactose mal-digestion (EFSA journal, 2010). In Humans, only a low proportion of population retains the capacity to degrade the lactose into adulthood, because the intestinal enzyme lactase-phlorizin hydrolase (more often called lactase or β-galactosidase) activity commonly starts decreasing after the first months or years of life [7], [8]. The benefits of yogurt are mainly linked to the metabolic capacities of *L. bulgaricus* and *S. thermophilus* that compensate the deficiency of the intestinal enzymes by their own β-galactosidase [9], [10], [11]. Our recent work brings concordant data about the preponderant role of metabolic adaptations of *S. thermophilus* in the gastrointestinal tract (GIT) of gnotobiotic rats. Previously, we have shown that *S. thermophilus* adapts its physiology to GIT by enhancing proteins devoted to carbohydrates metabolism [12]. It is likely that the overall glycolytic metabolic capacity of *S. thermophilus* is boosted in the GIT, thus explaining how the consumption of this viable microorganism may help the digestion of carbohydrates [9], [12]. In yogurt at the end of fermentation process, there is a significant amount of lactose (4–5%) [8], [13]. Thus, it could be hypothesized that residual lactose present in yogurt may be deleterious for lactose intolerant individuals; however, the inverse has been observed. The residual lactose in yogurt can be considered as a prebiotic for people with lactose mal-digestion [14], [15], [16]. The main mechanisms involved are linked to the adaptation of microbiota activity/composition in the presence of lactose, as illustrating by an enhanced faecal β-galactosidase activity [10], [15]. Concordantly, in gnotobiotic rats, β-galactosidase activity of *S. thermophilus* is higher in the GIT after lactose supplementation [17]. Thus the lactose-related metabolism of viable bacteria present in a final dairy product, and their transit throughout the GIT, is central to better understanding the beneficial effects of fermented products on health [18].

Although *S. thermophilus* can metabolize a number of different sugars (sucrose, glucose, fructose), lactose remains its favorite substrate. Lactose transport, metabolism, and regulation have been extensively studied *in vitro* by using different strains of *S. thermophilus* [19], [20] and in regard to their genomic sequences [21], [22]. In *S. thermophilus*, lactose is transported into the cell *via* the LacS permease which operates as a lactose/galactose antiporter or as a galactoside/H+ symporter [23]. LacS activity leads to the release of galactose into the extracellular medium after β-galactosidase has hydrolysed lactose into glucose and galactose. On the one hand, *S. thermophilus* is the archetype of lactose-adapted bacterium and on the other hand, we have observed that this specialized bacterium revealed a high adaptability of its carbohydrate metabolic pathways to the gut environment [12]. To this end, the objective of this work was to study the impact of lactose and lactose permease on *S. thermophilus* physiology in the GIT of gnotobiotic rats. We studied the sequenced strain LMD-9 for its colonization capacities, lactate production, proteomic profile and the related host responsiveness when animals received 4.5% of lactose that corresponded milk content of lactose. This work sheds new light on the lactose-related metabolism of *S. thermophilus* in the GIT and the subsequent crosstalk with colonic epithelium. By comparing the wild-type *S. thermophilus* strain and a mutant strain inactivated in lactose permease LacS, we also studied the involvement of its transporter in the carbohydrate metabolism *in vitro* and *in vivo*. Our results led us to propose a metabolic model of management of carbohydrates resources by *S. thermophilus in vivo.*

## Results

### Colonization and metabolic activity of *S. thermophilus* in the GIT and effects on colon epithelium

**Figure 1:**
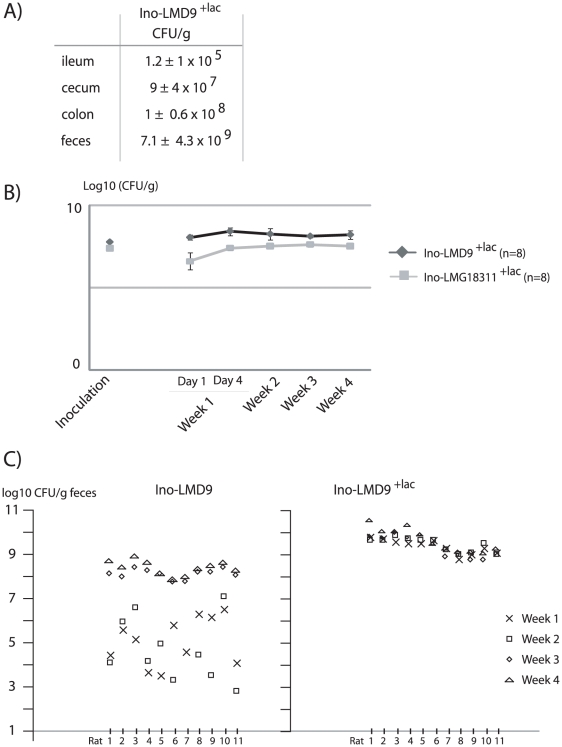
Colonization of *S. thermophilus* in mono-associated rats. One ml of *S. thermophilus* LMD-9 strain (6×10^8^ CFU) was inoculated in germ free rats thus leading to Ino-LMD9^+lac^ rats, which received water with lactose (4.5% wt/vol). Counts of *S. thermophilus* were obtained by plating on M17 agar lactose the luminal content from ileum, caecum, colon, faeces of Ino-LMD9^+lac^ (n = 11) rats 30 days after the inoculation. B) Log 10 CFU/g of *S. thermophilus* LMD-9 and LMG18311 counted from faecal samples all along the experiment for each mono-associated rat. The colonization curves obtained with LMG18311 and LMD-9 were not significantly different (p = 0.4). C) Comparison of colonization profile of LMD-9 when rats received lactose (Ino-LMD9^+lac^) or not [Ino-LMD9, data from our previous work [12]]. Each column symbolized one rat and X, □, ⋄, ▵ represented means of *S. thermophilus* LMD-9 counts obtained from faecal samples recovered, respectively, during the first, second, third and fourth week.

Germ free rats were orally inoculated with 6×10^8^ CFU of *S. thermophilus* LMD-9 and received lactose (4.5% wt/vol) in the drinking water (Ino-LMD9^+lac^) (Fig. 1). *S. thermophilus* was present along the GIT with an increasing amount of bacteria from the ileum to the colon (Fig. 1A). The maximum level of colonization for *S. thermophilus* occurred within the first week after gavage and this was also observed with another strain of *S. thermophilus*, LMG18311 strain (Ino-LMG18311^+lac^) (Fig. 1B). This rapid and high level of colonization was in sharp contrast with the progressive and lower final colonization observed when *S. thermophilus* was inoculated in rats that did not receive lactose (Fig. 1C) and [12].

L-lactate - an end product of *S. thermophilus* metabolism - accumulated at a 49.9±5.7 mM concentration in Ino-LMD9^+lac^ caecal content, that represented 20 fold higher L-lactate than in GF^+lac^ (2.5±1.5 mM). D-lactate was not detected in all groups of rats assessed. In parallel to this luminal L-lactate accumulation, a significant (p<0.05) acidification was noted with a luminal pH of 6.5±0.1 in Ino-LMD9^+lac^ when compared to 7.4±0.5 in GF^+lac^. In Ino-LMG18311^+lac^, 55.8±4.2 mM L-lactate accumulated in caecal content. Thus, *S. thermophilus* – either LMD-9 or LMG18311 strain - when colonizing the GIT was metabolically active by producing a high amount of L-lactate.

**Figure 2:**
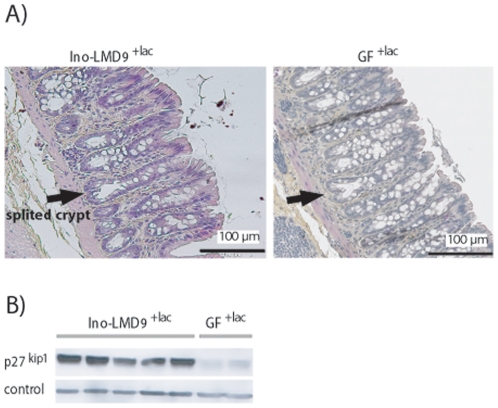
Colon epithelium of Ino-LMD9^+lac^. A) Representative photomicrograph represents a slide of a colon crypt from Ino-LMD9^+lac^ and GF^+lac^ stained with HES. The arrow represented crypt fission. B) Representative Western blot detected p27^kip1^ and a loading control (GAPDH). The total proteins of colon epithelium were from Ino-LMD9^+lac^ (n = 5) and GF^+lac^ (n = 2).

The global structure of Ino-LMD9^+lac^ epithelium was analysed by HES staining (Fig. 2A). The mean depth of colon crypt in Ino-LMD9^+lac^ was 206±9 µm that was not statistically different from values obtained with Ino-LMG18311^+lac^ (221±4 µm), GF^+lac^ (204±17 µm) and GF [24]. As illustrated, colon epithelium of Ino-LMD9^+lac^ was characterized by large and slack crypts and by the presence of crypts which seems to be ready to split (crypt fission). This global aspect of epithelium was reminiscent of our previous observations with various mono-associated and germ free rats [24]. We have previously proposed that lactate production from the metabolic activity of *S. thermophilus* stimulates an increases in monocarboxylic acid transporters mRNAs (SLC16A1 and SLC5A8) and a lactate-sensitive protein involved in the cell cycle arrest (p27^kip1^) in colon [12]. SLC16A1 and SLC5A8 mRNAs were respectively 1.8 (±0.05) and 1.6 (±0.3) induced in Ino-LMD9^+lac^ (n = 5) relative to GF^+lac^ (n = 3). p27^kip1^ protein was 1.8 (±0.03) induced in Ino-LMD9^+lac^ in comparison with GF^+lac^ (Fig. 2B), thus confirming that lactate may serve as a biological signal to communicate with host epithelium.

### The sugar utilisation of *S. thermophilus* is preferentially induced *in vivo*

We compared the proteomic profiles of *S. thermophilus,* by bi-dimensional electrophoresis, after growth in milk (inoculum solution) and after 30 days residence in the GIT (faeces of Ino-LMD9^+lac^) (Figure S1). Sixty-three proteins displayed different abundances between the 2 conditions: 25 were up-regulated (induction from 2 to 12 fold) and 38 were down-regulated (repression from 2 to 10 fold) in faeces compared to milk (Table 1).

**Table 1:** Functional distribution of *S. thermophilus* LMD-9 proteins whose abundance changed between the milk inoculum used for gavage and the faeces of Ino-LMD9^+lac^ rats (2-DE analysis).

	Functional category(number of proteins)	Range of modulation
25 up-regulated proteins in the digestive tract	-Carbohydrate metabolism: 16-Stress and fitness factors: 4-Nitrogen metabolism: 2-Nucleotide metabolism: 3	2.1–10.62.8–12.62.8–4.62.2–4.1
38 down-regulated proteins in the digestive tract	-Transcription/translation: 17-Diverse: 9- Nitrogen metabolism: 6- Nucleotide metabolism: 5-Carbohydrate metabolism: 1	2–102.2–102.5–52.5–102

Down-regulated proteins were mainly involved in transcription, translation, nitrogen metabolism (AA biosynthesis, peptidases), and purine and pyrimidine metabolisms. Thus, the *S. thermophilus* population was maintained at a high level (10^9^ CFU/g) in the GIT even with a low expression level of these pathways, whereas its growth in milk required a high expression and availability of these enzymes.

Sixteen out of twenty-five proteins up-regulated in Ino-LMD9^+lac^ faeces, compared to milk alone, belong to the carbohydrate metabolism (Figure S1 and Table 1). Of these 16 proteins, five were glycolytic enzymes (PfkA, Fba, GapA1, Eno, Pyk) while the others were “upstream” enzymes, (leading to the formation of compounds feeding the glycolytic pathway) and “downstream” enzymes (dissipating the ultimate product of glycolysis, pyruvate). Interestingly, enzymes potentially involved in metabolism of glycogen (GlgP, MalQ), sucrose (PtsK, ScrB), galactose (GalT, GalE), maltose (MalQ) or in polysaccharide synthesis (GalE) were significantly more abundant in the GIT than in milk (Figure S1). In comparison with a culture in milk, *S. thermophilus* ^+lac^ diversified its carbon source utilization by inducing the metabolism of alternative sugars in the GIT.

### The presence of lactose strengthens the sugar utilisation *in vivo*

**Figure 3:**
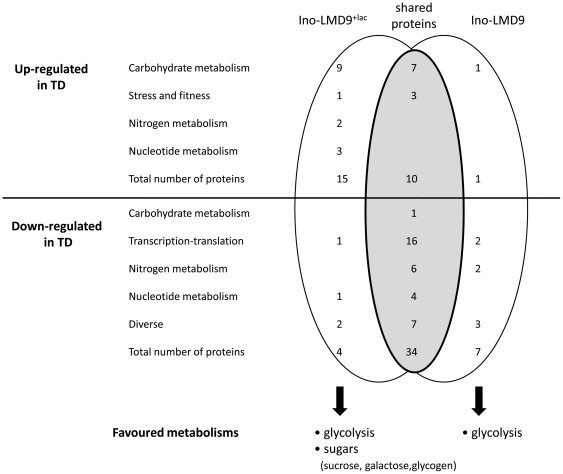
Comparative proteomic analysis (2-DE) of *S. thermophilus* LMD-9. Cytoplasmic extracts of *S. thermophilus* LMD-9 after passage through the GIT (ino-LMD9^+lac^ rats) were compared with those obtained in milk culture (inoculum for gavage). Data obtained in absence of lactose (Ino-LMD9) were previously described [12].

To study the effect of lactose on metabolic pathway of *S. thermophilus*, we compared two sets of data: i) Primarily listing the proteins differentially expressed in the Ino-LMD (without lactose) digestive tract *versus* milk and ii) a second listing of proteins differentially expressed in the Ino-LMD9^+lac^ digestive tract *versus* milk (Fig. 3). As soon as *S. thermophilus* was growing in the digestive tract, a large amount of proteins were down-regulated (38 and 41, in presence or absence of lactose, respectively). Thirty four of these intestine-repressed proteins that were both repressed in presence or absence of lactose (Fig. 3) were involved in staple functions as transcription and translation. These results confirmed that a low level of these proteins was sufficient to allow *S. thermophilus* to grow in the GIT and establish a metabolically active population.

Of the intestine-boosted proteins, 7 were involved in glycolysis and over-expressed either in the presence or in the absence of lactose (Fig. 3). General- (DnaK, GroEL) or oxidative-stress proteins (SodA) as well as the elongation factor Tuf –also potentially involved in adhesion/colonization- were more abundant in the GIT than in milk suggesting that they are related to environmental signal responses [11].

**Figure 4:**
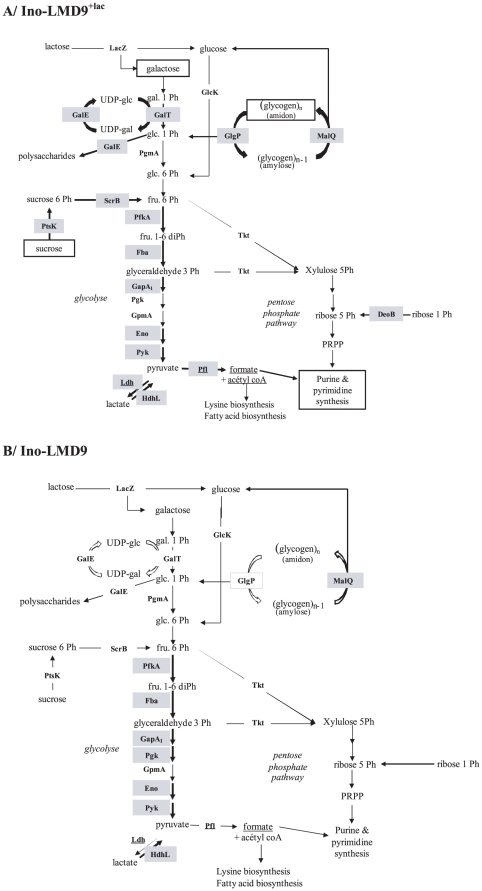
Model of carbohydrate metabolism of *S. thermophilus* in the digestive tract. Carbohydrate metabolism in *S. thermophilus* LMD-9 after its passage through the GIT, A) in presence (Ino-LMD9^+lac^) or B) in absence of lactose (Ino-LMD9). Up-regulated enzymes in GIT compared to milk are grey shaded. We constructed this model from the present proteomic data and from predictions of metabolism pathways of LMD-9 strain genome.

In the presence of lactose, 9 supplementary proteins involved in carbohydrate metabolism were over-expressed in the digestive tract (Fig. 3). The presence of lactose allowed *S. thermophilus* to enhance and diversify the metabolism of diverse carbohydrate sources, potentially explaining how the population of *S. thermophilus* overwhelms 10 fold the implanted population in absence of lactose (Fig. 1C). Based on the colonization curve, the proteomic signature and the resultant lactate end-product, the mobilization of the carbohydrate metabolism is determinant and limiting step for the colonization, the growth and the activity of *S. thermophilus in vivo* (Fig. 4).

### Effect of lactose permease LacS inactivation on the *S. thermophilus* metabolism

**Figure 5:**
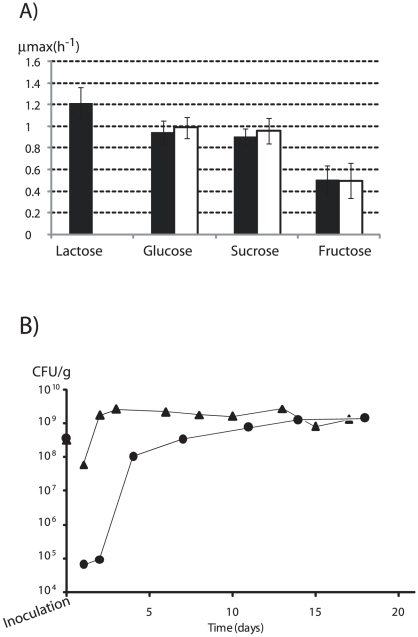
*S. thermophilus* Δ*lacS* characterization. A) *in vitro*: growth of the wild-type (▪) and the *ΔlacS* (□) strains in M17 in presence of different sugars. B) *in vivo*: enumeration of viable wild-type (▴) and *ΔlacS* (•) strains isolated from faeces of Ino-LMD9^+lac^ (n = 5) and Ino-Δ*lacS* ^+lac^ (n = 5), respectively.

In *S. thermophilus*, lactose is transported into the cell *via* the LacS permease which operates mainly as a lactose/galactose antiporter. Considering the central role of *S. thermophilus* carbohydrates metabolism *in vivo*, a negative mutant for the *lacS* gene was generated by inserting a kanamycine resistance cassette in the *lacS* gene. The resulting Δ*lacS* strain was validated *in vitro*, since it did not grow in the presence of lactose in contrast to the wild-type LMD-9 strain (Fig. 5A). In the presence of glucose, sucrose or fructose, Δ*lacS* and the wild-type LMD-9 strains displayed similar growth rates. *In vitro*, the inactivation of LacS permease impaired the growth in presence of lactose, while preserving the capacity of utilising other carbon sources.

The Δ*lacS* strain was then challenged *in vivo* for its capacity to colonize the GIT in germ-free rats receiving 4.5% lactose (Ino-Δ*lacS* ^+lac^ rats). The final level of colonization was identical with Δ*lacS* and LMD-9 strains, with 1.5 10^9^ CFU/g (Fig. 5B). Concordantly, the final amount of faecal lactate was identical in both lots of rats (30±0.5 mM in Ino-Δ*lacS* ^+lac^ *versus* 34.1±7 mM in Ino-LMD9^+lac^). However, the kinetic of colonisation was altered with Δ*lacS* strain in the first days after inoculation as the maximal colonization Δ*lacS* being delayed in comparison with the wild-type strain. In concordance with this delayed colonisation, the L-lactate was undetectable in the first days after inoculation in Ino-Δ*lacS* ^+lac^ faeces, whereas it was already high (41±12 mM) in faeces of Ino-LMD9^+lac^. These results indicated that LacS permease inactivation delayed the colonisation of *S. thermophilus* and suggested that an adaptation process occurred during the first 15 days after inoculation.

## Discussion

Our study demonstrates that the presence of lactose enhanced the fermentative activity of *S. thermophilus* leading to higher level of luminal lactate (×3.5 caecal lactate in Ino-LMD9^+lac^ compared to Ino-LMD9 [12]) which subsequent acts to modulate the host epithelium. Therefore, activation of enzymes involved in carbohydrate metabolism constitutes the metabolic signature of *S. thermophilus* in the GIT and allows the dialogue with colon epithelium. Lactose boosted the *S. thermophilus* carbohydrates metabolism that is already high in the digestive tract. *S. thermophilus*, probably because of its intensive use in dairy industry, has evolved by a specialization to lactose degradation with a genome that is considered as “poor”, having lost many genes. The sugar metabolism appears here to have key functions in adaptation to the GIT environment since life cycle of *S. thermophilus* in the GIT relies on carbon metabolism.

Recently, it has been shown that the efficiency of carbohydrates consumption by bifidobacteria correlated with their ability to protect host against infection [25]. From indigenous gut microbiota to sub-abundant transient species, the carbohydrate metabolism is central for bacterial colonization, activity and subsequent dialogue with host [26], [27], [28], [29], [30]. Although *S. thermophilus* is rather specialized in the degradation of simple sugars and belongs to numerically minor group of microbiota [31], its carbohydrate metabolism is also prevalent in regulating its own adaptation in GIT and its dialogue with host.

The present work confirmed our previous data stating that lactate produced in Ino-LMD9 by *S. thermophilus* induced both mRNAs SLC16A1/SLC5A8 monocarboxylic transporters and a cell-cycle arrest protein p27^kip1^ [12]. The levels of induction between Ino-LMD9^+lac^ and GF^+lac^ were not statistically different to that obtained between Ino-LMD9 and GF [12], indicating that the 3.7 fold highest content of lactate in Ino-LMD9^+lac^ (compared to Ino-LMD9) did not correlate with higher stimulation of epithelial transporters and p27^kip1^. All these results highlight the homeostatic responsiveness of the epithelium, as we have previously observed after intestinal resection [32]. In mono-associated rats, it is likely that a small portion of lactate was shuttled inside colonic cells, while the remaining luminal lactate was excreted in faeces (49.9±5.7 mM caecum *versus* 34.1±7 mM in faeces). In such models, the major proportion of lactate was excreted, because of the absence of other bacteria that normally inhabit the gut and metabolize lactate. In healthy human adults, lactate produced by gut microbiota is nearly undetectable in faecal samples since it is absorbed by host and also consumed by lactate-utilizing bacteria to produce mainly butyrate [33], [34], [35], [36]. Therefore, one can suppose that, in a context of a complex microbiota, *S. thermophilus* could contribute to microbial ecosystem by favoring lactate-utilizing enzymes or inhibiting low-pH sensitive bacteria [6].

Our study presents data on *S. thermophilus* physiology and demonstrates the flexibility of this bacterium to adapt to digestive environmental constraints and its capacity to diversify the use of alternative sugars. In absence of lactose, *S. thermophilus* develops a capacity to use alternative sugars *in vivo* ant this suggests that similar mechanisms occurred in the absence of a functional LacS permease. The presence of lactose enhanced LMD-9 colonization in the GIT, probably by favoring the use of diverse sugars in the GIT *via* the induction of metabolisms of galactose, sucrose, maltose, and glycogen. Each metabolic pathway feeds glycolysis, suggesting that *S. thermophilus* metabolism converges to ATP production in the GIT. This also indicates that most of the sugar routes predicted by genome analysis, and so far not yet studied, are probably functional in *S. thermophilus* LMD-9. Δ*lacS* strain needed about 15 days to stably and maximally implant and thus possibly to set up a metabolic response by using alternative sugars and/or by favoring the entrance of lactose by other unknown transporters. By studying the physiology of *S. thermophilus* in the digestive tract, we also propose that the protein MalQ may be involved in glycogen synthesis rather than in maltose metabolism. In absence of lactose, MalQ was induced in gut and we hypothesized that *S. thermophilus* stocked glycogen in order to face limiting environmental conditions [37]. In presence of lactose, both MalQ and GlgP [responsible for glycogen breakdown in bacteria [38]] were induced, suggesting that equilibrium between anabolism and catabolism occurs in *S. thermophilus*. A similar simultaneous glycogen synthesis and degradation have been described in *Corynebacterium glutamicum* where glycogen is constantly recycled [39]. All our observations show that MalQ is not involved in maltose metabolism, in concordant with the fact that no MalE transporter is present in the genome of LMD-9 and that genes *malQ* and *glgP* (encoding potentially for a glycogen phosphorylase) are an operon in LMD-9. Glycogen could be of prime importance in the colonization, adaptation and survival of *S. thermophilus* within the GIT, as previously observed for other microorganisms, when present in starvation conditions [37] or during transition between nutrient-rich- and nutrient-poor environments [40].

Our present work sheds new light on the established association of *S. thermophilus* and lactose by revealing that lactose enhanced *S. thermophilus* kinetics, level of colonization and fermentative activity in the GIT. Our work suggests that a food product containing both a live *S. thermophilus* and lactose would favor the colonization and fermentative activity of *S. thermophilus in vivo*. In this context, our results follow the rationale of previous clinical observations and the nutritional assertion that the consumption of yogurt (containing lactose) could alleviate the symptoms of lactose intolerance.

## Materials and Methods

### Bacterial strains, media, and inoculating samples

The strains *S. thermophilus* LMD-9 (ATCC BAA-491, USA), and LMG18311 (BCCM collection, Belgium) were used. Stock cultures of *S. thermophilus* LMD-9 and LMG18311 were prepared in reconstituted 10% (wt/vol) Nilac skim milk (NIZO, Ede, the Netherlands) as previously described [41]. *S. thermophilus* monocultures were obtained by inoculating Nilac milk with 10^6^ CFU/ml of stock cultures and incubated at 42°C until pH 5.4–5.5. One ml of culture was used for rat gavage and the remaining aliquots were frozen in liquid nitrogen and stored at −20°C until protein extraction. The cultures were enumerated *a posteriori* by plating appropriate dilutions on M17 agar lactose (10 g/L) for *S. thermophilus*. After 16 h (*S. thermophilus*) incubation at 42°C under anaerobiosis (Anaerocult A, Merck, Darmstadt, Germany), colonies were counted.

### μ_max_ determination

Cultures (n = 3) of *S. thermophilus* strains were performed in M17 supplemented with 10 g/L lactose, glucose, sucrose or fructose; the apparent growth rate (μ_max_) was defined as the maximum slope of semi-logarithmic representation of growth curves assessed by O. D._650 nm_ measurements.

### Insertional inactivation of *S. thermophilus* LMD-9 *lacS* gene

The kanamycin cassette of the plasmid pKa 2000 was PCR-amplified using the Phusion high fidelity DNA polymerase with AphA3-F ( ^5′^CCAGCGAACCATTTGA^3′^ ) and AphA3-R ( ^5′^GTTGCGGATGTACTTCAG^3′^ ) primers. The 1489 bp DNA fragments flanking the *lacS* gene were PCR-amplified using the Phusion DNA polymerase, the LMD-9 DNA as a template, and primers LacS-up ( ^5′^TATGTGCCTGCCAGTCCA^3′^ )/Kana-up-R ( ^5′^AGGGGTCCCGAGCGCCTACGAGGAATTTGTATCGATGACCTTTAGATTTTTCCAT^3′^ ) for the upstream fragment and primers LacS-down ( ^5′^AACTGGAACGACTTCAAC^3′^ )/Kana-down ( ^5′^CTTACCTATCACCTCAAATGGTTCGCTGGGTTTATCGTAACTAATTCAGAAAAA^3′^ ) for the downstream fragment. The 3′ end of the upstream generated fragment contained a sequence complementary to the 5′ end of the *kana* cassette whereas the 5′ end of the downstream generated fragment contained a sequence complementary to the 3′ end of the cassette. This allowed joining of these three fragments subsequent to Taq Phusion PCR using primers LacS-up and LacS-down. After purification with a QIAquick PCR purification kit, 500 ng of the resulting 3.4 kb fragment was further used to transform LMD-9 natural competent cells as described by Gardan *et al*. [42]. Transformants were selected on M17Glu plates with kanamyin and were then checked by PCR using oligonucleotides LacS-up and KanaR. Finally sequencing of the flanking regions was performed to ensure that no unwanted mutations were introduced.

### Animals and experimental design

All procedures were carried out in accordance with European and French guidelines for the care and use of laboratory animals. Permission 78–123 is a permit number dedicated to M. Thomas. MICALIS (Microbiologie de l'Alimentation au Service de la Santé) review board specifically approved this study. At the age of 2 months, germ-free (GF) rats (male, Fisher 344) were inoculated either with *S. thermophilus* LMD-9 (Ino-LMD9^+lac^, n = 11) or *S. thermophilus* LMG18311 (Ino-LMG18311^+lac^, n = 8). 1 ml of a culture of *S. thermophilus* in Nilac milk (5×10^8^ CFU/ml) was transferred to GF rats by oral gavage. As a control, GF^+lac^ rats were also inoculated with 1 ml of sterile Nilac milk (without bacteria). Following gavage, rats received water enriched with lactose (4.5%wt/vol). GF and mono-associated rats were housed in sterile Plexiglas isolators (Ingénia, Vitry-sur-Seine, France). All groups of rats received the same standard diet (UAR), which was sterilized by gamma irradiation. Twice a week *S. thermophilus* was enumerated by plating serial dilutions of the faeces on M17 lactose agar. All rats were euthanized at three months old, 30 days after gavage.

*S. thermophilus* Δ*lacS* strain was inoculated in GF rats to obtain Ino-Δ*lacS^+lac^* rats. The Δ*lacS* inoculum was grown in M17+glucose and bacteria were enumerated by plating serial dilutions of the faeces on M17 glucose agar supplemented with kanamycine (1 mg/ml). Ino-Δ*lacS* ^+lac^ (n = 5) rats drank 4.5% lactose-enriched water. The presence of kanamycin cassette disrupting the *lacS* gene was checked by PCR in faeces of Ino-Δ*lacS* ^+lac^. Ino-Δ*lacS* ^+lac^ rats were euthanized 18 days after gavage.

Rats were anesthetized with isoflurane and tissues were recovered. The colon was immediately used, for colonic epithelial cell isolation or for histological procedures as it has been described in Cherbuy *et al.* [43], [44].

### Western blot analysis

Colonic proteins from isolated epithelial cells were used for Western blot analysis as previously described [24] by using a denaturing (SDS)–polyacrylamide gel. Proteins were analysed using anti- p27^kip1^ (Santa Cruz Biotechnology; 1/500). GAPDH was used as loading control. Signals imprinted on autoradiography films were quantified by scanning densitometry of the autoradiograph using Biovision 1000 and logiciel bio1D (Vilber Lourmat, France).

### Dosage of d- and l-lactates

d- and l-lactates were measured in caecal contents and faeces with the Biosentec d/l lactic acid enzymatic kits according to the manufacturer instructions (Biosentec, Toulouse, France) as also described in Rul *et al*. [12].

### Histology analysis

Colon samples were cut into 2 cm sections, fixed in 4% paraformaldehyde (4 hours, room temperature), dehydrated and embedded in paraffin according to standard histological protocols. Four micrometer sections were mounted on SuperFrost® Plus slides. Slides were stained with Hematoxylin-Eosin-Safran (HES) for histological analysis. Crypt depths were determined with NDP.view software (Hamamatsu). Only U shaped longitudinally cut crypts with open lumina along the crypt axis were analysed. Results were the mean obtained by analysis at least 20 crypts per rat (Ino-LMD9^+lac^, n = 5; Ino-LMG18311^+lac^, n = 3).

### RNA isolation and quantitative RT-PCR analysis

Total RNAs were extracted from colon epithelial cells of GF^+lac^ (n = 3), and Ino-LMD9^+lac^ (n = 5) by the guanidinium thiocyanate method. The *slc16a1* and slc*5a8* mRNA quantification was performed as we have previously described [12].

### Bacterial protein extraction, comparative bi-dimensional (2-DE) protein analysis and image analysis

Cytoplasmic proteins were extracted from bacteria as previously described [12] either from milk cultures (n = 3) or faecal samples (5 g of frozen faeces from 3 Ino-LMD9^+lac^ rats). A volume of cytosolic fraction corresponding to 250 µg of proteins was treated as previously described [45]. Bi-dimensional (2-DE) protein analysis and image analysis were performed according to Rul *et al.* [12]. MS analyses were performed using a Voyager-DE-STR (Applied Biosystems, Framingham, USA) on the PAPPSO proteomic platform (http://pappso.inra.fr). The proteins were identified using MS-FIT (http://prospector.ucsf.edu).

### Statistical analysis

Results are presented as means ± SE for the number of animals indicated. Comparisons of group data between different batches of rats were performed using one–way analysis of variance (ANOVA) followed by Tukey's student range test where appropriate. Significance was for *P* value lower than 0.05. Statistical analysis was performed using the JMP® software (version 7, SAS institute INC).

## Supporting Information

Figure S1 Fold changes in protein abundance (2-DE) of *S. thermophilus* LMD-9 between faeces in presence of lactose and late growth phase in milk.
(DOCX)
