# Supplementary material for: Carbohydrate Metabolism Is Essential for the Colonization of Streptococcus thermophilus in the Digestive Tract of Gnotobiotic Rats
Source: PLoS One. 2011 Dec 22;6(12):e28789. doi: 10.1371/journal.pone.0028789 (PMC3245227; doi:10.1371/journal.pone.0028789)
Supplement: Figure S1 — Fold changes in protein abundance (2-DE) of S. thermophilus LMD-9 between faeces in presence of lactose and late growth phase in milk. (DOCX) [file pone.0028789.s001.docx]

Gene/locus pI_th_ MW_th_ Peptide Sequence Fold Protein

(kDa) number coverage (%) change identification

**Higher level in TD**

**Carbohydrate metabolism**

*deoB* 4.91 44.4 12 37 3.6 Phosphopentomutase

*eno* 4.67 47 22 63 6.1 Phosphoenolpyruvate carboxylase

*galE1* 5.12 36.8 9 36 6.8 UDP-glucose 4 epimerase

*galE2* 4.96 37.2 10 33 2.1 UDP-glucose 4-epimerase

*galT* 4.75 56.5 11 29 6.9 Galactose-1-phosphate

uridyltransferase

*gapA1* 5.26 36 9 30 3.1 Glyceraldehyde-3-phosphate

dehydrogenase

*glgP* 5.36 86.5 12 18 10.6 Glucan phosphorylase

*hdhL* 5.09 34.4 11 41 2.4 Putative L-2-hydroxy

isocaproate/malate/lactate

dehydrogenase

*ldh*  4.67 35.4 6 26 2.1 L-lactate dehydrogenase

*malQ* 5.06 56.6 11 19 4.2 4-alpha-glucanotransferase

*pfkA* 4.87 36 17 43 4.8 6-phosphofructokinase

*pfl* 4.99 87 9 15 4.9 Pyruvate-formate lyase

*ptsK* 5.18 34.3 10 36 2.4 Serine kinase of the HPr protein

*pyk* 4.95 54.5 13 31 2.4 Pyruvate kinase

*scrB* 4.87 54.4 17 43 5 Sucrose-6-phosphate hydrolase

*fba* 4.81 31.5 9 47 2.8 Fructose/tagatose bisphosphate

aldolase

**Stress**

*dnaK*  4.62 64.8 18 42 6.3 Molecular chaperone

*sodA* 4.82 22.5 6 48 2.8 Superoxide dismutase (Mn)

*groEL* 4.7 56.9 35 69 11.1 Chaperonin GroEL (HSP60 family)

**Translation**

*tuf*  4.9 43.9 15 45 12.6 GTPase - translation elongation factor

**Nucleotide metabolism**

*punA* 5.45 28.8 10 52 4.1 Purine nucleoside phosphorylase

*ster_0303* 4.86 36.1 8 33 2.2 Xanthosine triphosphate

pyrophosphatase

*upp* 5.45 23.1 12 55 2.9 Uracil phosphoribosyltransferase

**Nitrogen metabolism**

*asnA* 5.1 37.5 13 31 2.8 Asparagine synthetase A

*pepX* 5.2 85.7 18 30 4.6 X-prolyl dipeptidylaminopeptidase

**Lower level in TD**

**Transcription, translation**

*alaS* 5.03 96.7 25 31 0.2 Alanyl-tRNA synthetase

*asnS* 4.93 51.1 27 51 0.3 Asparaginyl-tRNA synthetase

*aspS* 4.81 65.9 21 37 0.2 Aspartyl-tRNA synthetase

(GTPase)

*gltX* 4.97 55.3 26 47 0.5 Glutamyl- and glutaminyl-tRNA

synthetase

*glyQ* 4.91 34.9 9 33 0.4 Glycyl-tRNA synthetase, alpha

subunit

*glyS* 5.05 78.5 22 38 0.2 Glycyl-tRNA synthetase, beta subunit

*leuS* 4.99 94.4 13 17 0.2 Leucyl-tRNA synthetase

*pheT* 4.83 88.6 24 36 0.2 Phenylalanyl-tRNA synthetase beta

chain

*rplJ* 5.36 17.5 7 43 0.3 Ribosomal protein L10

*rplL* 4.42 12.4 9 81 0.1 Ribosomal protein L7/L12

*rpoB* 5.02 29.2 13 50 0.4 DNA-directed RNA polymerase, beta

subunit/140 kD subunit

*rpsA* 4.88 43.9 21 55 0.3 Ribosomal protein S1

*rpsB* 5.3 28.4 12 43 0.2 30S ribosomal protein S2

*ster_1166* 5.02 29.2 13 50 0.4 Putative translation factor

*thrS* 5.39 74.8 36 45 0.2 Threonyl-tRNA synthetase

*tig* 4.41 46.7 22 53 0.3 FKBP-type peptidyl-prolyl cis-trans

isomerase (trigger factor)

*tsf* 4.75 37.4 15 60 0.1 Translation elongation factor Ts

**Nitrogen metabolism**

**Amino acids biosynthesis**

*bcaT* 4.9 37.5 9 30 0.2 Branched chain amino acid

aminotransferase

*cysM1* 5.2 32.3 15 51 0.2 Cysteine synthase

*lysA* 4.81 46.4 10 38 0.3 Diaminopimelate decarboxylase

*metK* 4.93 44.7 15 44 0.3 S-adenosylmethionine synthetase

**Peptidases**

*pepC* 4.85 50.4 9 17 0.4 Aminopeptidase C

*pepN* 4.59 96.3 15 19 0.3 Aminopeptidase N

**Nucleotide biosynthesis and**

**salvage**

*pyrG* 5.51 59.1 12 28 0.2 CTP synthase (UTP-ammonia lyase)

*purC* 5.00 27 22 80 0.3 Phosphoribosylaminoimidazole –

succinocarboxamide synthase

*purL* 4.93 136 19 19 0.4 Phosphoribosylformylglycinamidine

synthase II (FGAM synthetase)

*guaB* 5.31 52.9 13 29 0.1 IMP dehydrogenase/GMP reductase

*nrdF* 4,5 37 11 10 0.2 Ribonucleotide-diphosphate reductase

beta subunit

**Cell division protein**

*ftsZ* 4.6 46.5 17 43 0.4 Cell division GTPase

**Carbohydrate metabolism**

*gdhA* 5.21 48.3 19 60 0.5 Glutamate dehydrogenase/leucine

dehydrogenase

**Miscellaneous**

*atpA* 5.09 54.6 17 35 0.39 ATP synthase subunit A

*murC* 5.23 49.6 15 44 0.3 UDP-N-acetylmuramate-alanine

ligase

*panE* 5.6 36.1 13 42 0.4 Ketopantoate reductase

*pncB* 5.3 54.4 9 20 0.45 Nicotinic acid

phosphoribosyltransferase

*secA* 5.22 96.4 28 41 0.1 Preprotein translocase subunit SecA

*ster_0157* 4.77 46.4 15 38 0.4 Hypothetical protein

*ster_1854* 4.41 59.5 17 32 0.1 Predicted kinase related to

dihydroxyacetone kinase

*typA* 4.9 68.7 21 38 0.2 Predicted membrane GTPase

involved in stress response

|  |  |  |  |  |
| --- | --- | --- | --- | --- |
